# 5-Meth­oxy-*N*,*N*-di-*n*-propyl­tryptamine (5-MeO-DPT): freebase and fumarate

**DOI:** 10.1107/S2056989021003753

**Published:** 2021-04-13

**Authors:** Duyen N. K. Pham, Andrew R. Chadeayne, James A. Golen, David R. Manke

**Affiliations:** a University of Massachusetts Dartmouth, 285 Old Westport Road, North Dartmouth, MA 02747, USA; b CaaMTech, LLC, 58 East Sunset Way, Suite 209, Issaquah, WA 98027, USA

**Keywords:** crystal structure, tryptamines, indoles, hydrogen bonding

## Abstract

The solid-state structures of the synthetic psychedelic 5-meth­oxy-*N*,*N*-di-*n*-propyl­tryptamine (5-MeO-DPT) {systematic name: *N*-[2-(5-meth­oxy-1*H*-indol-3-yl)eth­yl]-*N*-propyl­propan-1-amine}, C_17_H_25_N_2_O, and its fumarate salt, bis­(5-meth­oxy-*N*,*N*-di-*n*-propyl­tryptammonium) fumarate (systematic name: bis­{*N*-[2-(5-meth­oxy-1*H*-indol-3-yl)eth­yl]-*N*-propyl­propan-1-aminium} but-2-ene­dio­ate), 2C_17_H_25_N_2_O^+^·C_4_H_2_O_4_^2−^, are reported. The freebase has a single tryptamine mol­ecule in the asymmetric unit. The mol­ecules are linked together by N—H⋯N hydrogen bonds in zigzag chains along the [010] direction. The fumarate salt has a single tryptammonium cation and half of a fumarate dianion in the asymmetric unit. The tryptammonium and fumarate ions are held together in one-dimensional chains by a series of N—H⋯O hydrogen bonds. These chains are combinations of *R* ^4^_4_(22) rings, and *C* ^2^_2_(14) and *C* ^4^_4_(28) parallel chains along [001].

## Abstract 2

The solid-state structure of the synthetic psychedelic 5-MeO-DPT is reported in its freebase form and as its fumarate salt.

## Chemical context   

5-Meth­oxy-*N*,*N*-di­methyl­tryptamine (5-MeO-DMT) is a psychoactive indole­alkyl­amine that is found in a number of plants and animals, but is best known to be present in the parotid glands of the Colorado River toad, *Bufo alvarius* (Shen *et al.*, 2010 ▸). 5-MeO-DMT demonstrates high activity at the serotonin (5-hy­droxy­tryptamine, 5-HT) 2A receptor, which leads to its psychotropic activity. Recent research has pointed to 5-MeO-DMT as a promising pharmaceutical in the treatment of mental health disorders (Uthaug *et al.*, 2019 ▸). There are a number of synthetic *N*,*N*-dialkyl derivatives including 5-meth­oxy-*N*-methyl-*N*-iso­propyl­tryptamin (5-MeO-MiPT), 5-meth­oxy-*N*,*N*-di­ethyl­tryptamine (5-MeO-DET), 5-meth­oxy-*N*,*N*-di-*n*-propyl­tryptamine (5-MeO-DPT) and 5-meth­oxy-*N*,*N*-diiso­propyl­tryptamine (5-MeO-DiPT). Alexander Shulgin described the experience associated with inhalation of these derivatives in humans, with 5-MeO-DMT described as ‘positive and out-of-body’ while 5-MeO-DPT was described as ‘good and bad’ (Shulgin & Shulgin, 1997 ▸). 5-MeO-DPT has not been described much in the scientific literature, though a recent report described its activity at the 5-HT_1A_ and 5-HT_2A_ receptors showing 75–100% of full agonism at both receptors (Åstrand *et al.*, 2020 ▸). As this class of compounds becomes more important for the treatment of mental health in humans, an in depth understanding of these compounds and how the structural changes impact the clinical experience in humans is going to be significant. To do so, it is important to have analytically pure, well-characterized compounds, ideally as crystalline materials. Herein we report the first structures of 5-MeO-DPT, both as its freebase and as its fumarate salt.
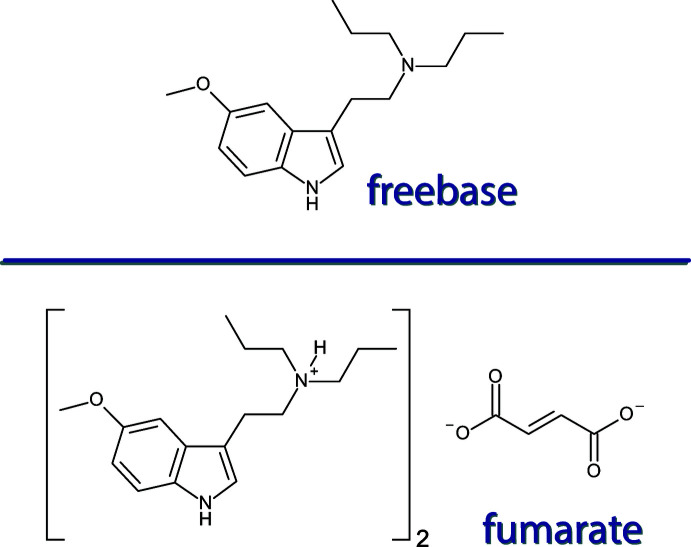


## Structural commentary   

**Figure 1:**
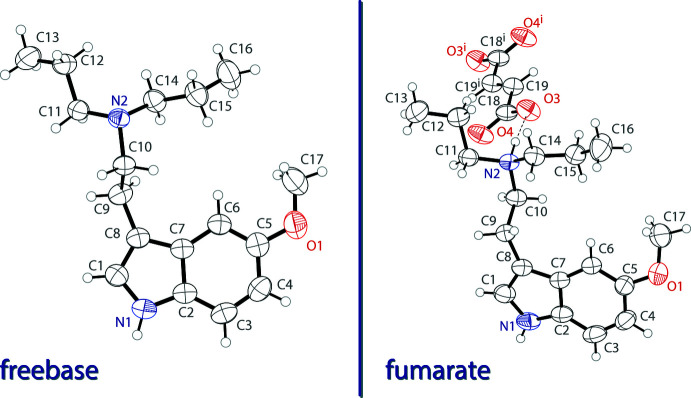
The mol­ecular structures of 5-MeO-DPT freebase (left) and 5-MeO-DPT fumarate (right), showing the atomic labeling. Displacement ellipsoids are drawn at the 50% probability level. Hydrogen bonds are shown as dashed lines. Symmetry code: (i) 1 − *x*, 2 − *y*, 2 − *z*.

The asymmetric unit of 5-meth­oxy-*N*,*N*-di-*n*-propyl­tryptamine (5-MeO-DPT) freebase contains a single tryptamine mol­ecule (Fig. 1 ▸, left). It possesses a near planar indole unit with an r.m.s. deviation from planarity of 0.012 Å. The meth­oxy group is in the same plane as the indole ring with a C6—C5—O1—C17 torsion angle of −1.2 (2)°. The ethyl­amino arm is turned away from the indole plane with a C1—C8—C9—C10 torsion angle of 110.4 (2)°.

The asymmetric unit of bis­(5-meth­oxy-*N*,*N*-di-*n*-propyl­tryptammonium) fumarate contains one tryptammonium cation and half of a fumarate dianion (Fig. 1 ▸, right). The tryptammonium cation possesses a near planar indole unit with a deviation from planarity of 0.015 Å. The meth­oxy group is turned slightly from the plane of the indole ring with a C6—C5—O1—C17 torsion angle of −13.5 (4)°. The ethyl­amino arm is turned away from the indole plane with a C1—C8—C9—C10 torsion angle of −104.8 (3)°. The second half of the fumarate dianion is generated by inversion, and is near planar with an r.m.s. deviation from planarity of 0.022 Å.

## Supra­molecular features   

**Figure 2:**
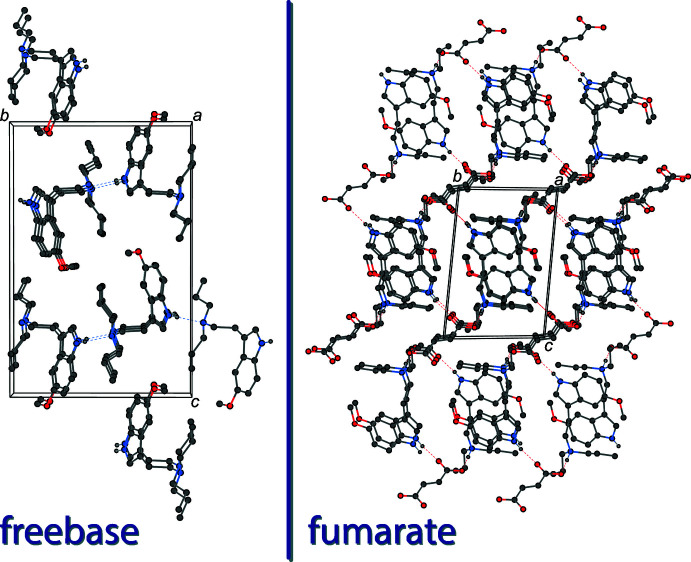
The crystal packing of 5-MeO-DPT freebase (left), viewed along the *a* axis, and the crystal packing of 5-MeO-DPT fumarate (right), viewed along the *a* axis. The hydrogen bonds (Tables 1 ▸ and 2 ▸) are shown as dashed lines. Hydrogen atoms not involved in hydrogen bonds are omitted for clarity.

In the solid-state structure of 5-MeO-DPT freebase, the mol­ecules are held together by an N1—H1⋯N2 hydrogen bond between the indole N—H and the amino nitro­gen atom. These hydrogen bonds join the mol­ecules together in infinite chains along the [010] direction (Table 1 ▸). The crystal packing of 5-MeO-DPT freebase is shown on the left in Fig. 2 ▸.

**Figure 3:**
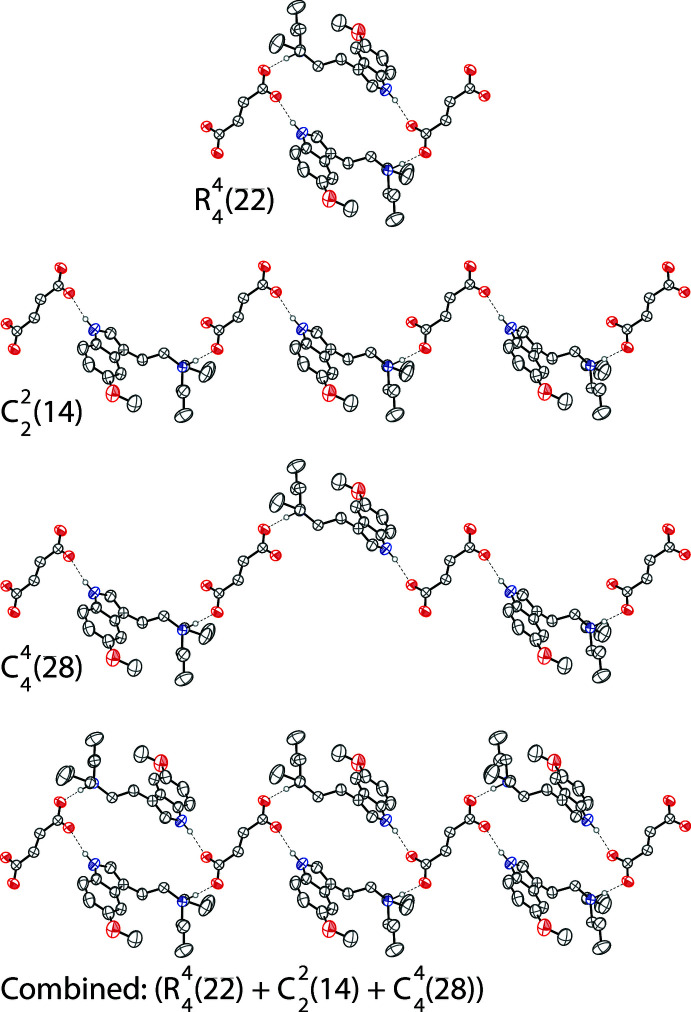
The hydrogen-bonding network along [001], which consists of 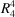
(22) rings that are joined together by two parallel 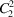
(14) and 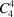
(28) chains. The three components described in graph-set notation and the combined chain is shown. Displacement ellipsoids are drawn at the 50% probability level. Hydrogen atoms not involved in hydrogen bonding are omitted for clarity. Hydrogen bonds are shown as dashed lines.

In the structure of 5-MeO-DPT fumarate, the tryptammonium cation is linked to the fumarate dianion in the asymmetric unit through an N2—H2⋯O3 hydrogen bond between the ammonium nitro­gen and a carboxyl­ate oxygen of the fumarate. There is also an N1—H1⋯O4 hydrogen bond between the indole nitro­gen and the other oxygen of the carboxyl­ate group on a symmetry-generated fumarate dianion (Table 2 ▸). The crystal packing of 5-MeO-DPT fumarate is shown on the right in Fig. 2 ▸. Two tryptammonium cations and two fumarate dianions are joined together through these hydrogen bonds to form rings with graph-set notation 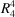
(22) (Etter *et al.*, 1990 ▸). The rings are joined together by two parallel chains along [001]. These chains have graph-set notation 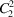
(14) and 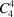
(28). The chains and rings are shown in Fig. 3 ▸.

## Database survey   

The two structures reported are most closely related to the freebase of 5-meth­oxy-*N*,*N*-di­allyl­tryptamine, or 5-MeO-DALT (CCDC 1995802; Chadeayne *et al.*, 2020*c* ▸), and the fumarate of 5-MeO-DALT (Pham, Sammeta *et al.*, 2021 ▸), which exhibit solid-state structures that are very similar to those reported here. The freebase of 5-MeO-DPT and 5-MeO-DALT have nearly identical unit cells. The fumarates of the two 5-MeO-DPT analogs exhibit the same chains, showing 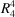
(22) rings and 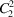
(14) and 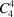
(28) chains in both cases. The other *N*,*N*-di-*n*-propyl­tryptamine structures known are 4-hy­droxy-*N*,*N*-di-*n*-propyl­tryptammonium chloride (Sammeta *et al.*, 2020 ▸) and bis­(4-hy­droxy-*N*,*N*-di-*n*-propyl­tryptammonium) fumarate (CCDC 1962339; Chadeayne, Pham *et al.*, 2019 ▸). The other tryptamine freebase structures known are the natural products *N*,*N*-di­methyl­tryptamine, or DMT (DMTRYP; Falkenberg, 1972 ▸), 5-MeO-DMT (QQQAGY; Bergin *et al.*, 1968 ▸), psilocybin (PSILOC; Weber & Petcher, 1974 ▸), psilocin (PSILIN; Petcher & Weber, 1974 ▸) and norpsilocin (CCDC 1992279; Chadeayne *et al.*, 2020*b* ▸), and the synthetic psychedelic *N*-methyl-*N*-*n*-propyl­tryptamine (WOHYAW; Chad­eayne *et al.*, 2019*b* ▸). The other fumarate salts of tryptamines known are norpsilocin (CCDC 1992278; Chadeayne *et al.*, 2020*b* ▸), 4-hy­droxy-*N*-methyl-*N*-iso­propyl­tryptamine (CCDC 1962339; Chadeayne *et al.*, 2020*a* ▸), 5-meth­oxy-2,*N*,*N*-tri­methyl­tryptamine (Pham, Chadeayne *et al.*, 2021 ▸) and psilacetin (HOCJUH; Chadeayne *et al.*, 2019*a* ▸).

## Synthesis and crystallization   

Slow evaporation of an acetone solution of a commercial sample (Chem Logix) of 5-MeO-DPT freebase resulted in the formation of crystals of 5-meth­oxy-*N*,*N*-di-*n*-propyl­tryptamine suitable for X-ray analysis. Crystals of bis­(5-meth­oxy-*N*,*N*-di-*n*-propyl­tryptammonium) fumarate were grown from the slow evaporation of an aceto­nitrile solution of a commercial sample (Chem Logix) of 5-MeO-DPT fumarate.

## Refinement   

Crystal data, data collection and structure refinement details are summarized in Table 3 ▸.

## Supplementary Material

Crystal structure: contains datablock(s) umd2187e_a, umd2188f_a. DOI: 10.1107/S2056989021003753/zq2261sup1.cif

Structure factors: contains datablock(s) umd2187e_a. DOI: 10.1107/S2056989021003753/zq2261umd2187e_asup2.hkl

Structure factors: contains datablock(s) umd2188f_a. DOI: 10.1107/S2056989021003753/zq2261umd2188f_asup3.hkl

Supporting information file. DOI: 10.1107/S2056989021003753/zq2261umd2187e_asup4.cml

Supporting information file. DOI: 10.1107/S2056989021003753/zq2261umd2188f_asup5.cml

CCDC references: 2075928, 2075927

Additional supporting information:  crystallographic information; 3D view; checkCIF report

## Figures and Tables

**Table 1:** Hydrogen-bond geometry (Å, °) for 5-MeO-DPT freebase[Chem scheme1]

*D*—H⋯*A*	*D*—H	H⋯*A*	*D*⋯*A*	*D*—H⋯*A*
N1—H1⋯N2^i^	0.878 (18)	2.167 (19)	3.0070 (17)	160.0 (16)

Symmetry code: (i) -x+{\script{3\over 2}}, y+{\script{1\over 2}}, -z+{\script{1\over 2}}.

**Table 2:** Hydrogen-bond geometry (Å, °) for 5-MeO-DPT fumarate[Chem scheme1]

*D*—H⋯*A*	*D*—H	H⋯*A*	*D*⋯*A*	*D*—H⋯*A*
N2—H2⋯O3	0.98 (2)	1.68 (2)	2.6588 (17)	175 (2)
N1—H1⋯O4^i^	0.86 (2)	1.91 (3)	2.757 (2)	171 (2)

Symmetry code: (i) -x+1, -y+2, -z+1.

**Table 3:** Experimental details

	5-MeO-DPT freebase	5-MeO-DPT fumarate
Crystal data		
Chemical formula	C_17_H_26_N_2_O	C_17_H_27_N_2_O^+^·0.5C_4_H_2_O_4_^2−^
*M*_r_	274.40	332.43
Crystal system, space group	Monoclinic, *P*2_1_/*n*	Triclinic, *P*\overline{1}
Temperature (K)	297	297
*a*, *b*, *c* (Å)	6.2223 (3), 13.0931 (6), 19.7791 (10)	9.2956 (6), 9.4443 (6), 12.7427 (8)
α, β, γ (°)	90, 91.825 (2), 90	78.552 (2), 75.929 (2), 60.806 (2)
*V* (Å^3^)	1610.57 (13)	943.06 (11)
*Z*	4	2
Radiation type	Mo *K*α	Mo *K*α
μ (mm^−1^)	0.07	0.08
Crystal size (mm)	0.38 × 0.3 × 0.06	0.3 × 0.22 × 0.2

Data collection		
Diffractometer	Bruker D8 Venture CMOS	Bruker D8 Venture CMOS
Absorption correction	Multi-scan (*SADABS*; Bruker, 2018 ▸)	Multi-scan (*SADABS*; Bruker, 2018 ▸)
*T*_min_, *T*_max_	0.687, 0.745	0.722, 0.745
No. of measured, independent and observed [*I* > 2σ(*I*)] reflections	29365, 3035, 2466	37231, 3565, 3006
*R*_int_	0.038	0.032
(sin θ/λ)_max_ (Å^−1^)	0.610	0.611

Refinement		
*R*[*F* ^2^ > 2σ(*F* ^2^)], *wR*(*F* ^2^), *S*	0.042, 0.117, 1.06	0.052, 0.142, 1.05
No. of reflections	3035	3565
No. of parameters	189	228
H-atom treatment	H atoms treated by a mixture of independent and constrained refinement	H atoms treated by a mixture of independent and constrained refinement
Δρ_max_, Δρ_min_ (e Å^−3^)	0.14, −0.17	0.29, −0.15

Computer programs: *APEX3* and *SAINT* (Bruker, 2018 ▸), *SHELXT2014* (Sheldrick, 2015*a* ▸), *SHELXL2018* (Sheldrick, 2015*b* ▸), *OLEX2* (Dolomanov *et al.*, 2009 ▸) and *publCIF* (Westrip, 2010 ▸).

## supplementary crystallographic information

### N-[2-(5-Methoxy-1H-indol-3-yl)ethyl]-N-propylpropan-1-amine (umd2187e_a) Crystal data

**Table:**

C_17_H_26_N_2_O	*F*(000) = 600
*M**_r_* = 274.40	*D*_x_ = 1.132 Mg m^−^^3^
Monoclinic, *P*2_1_/*n*	Mo *K*α radiation, λ = 0.71073 Å
*a* = 6.2223 (3) Å	Cell parameters from 9916 reflections
*b* = 13.0931 (6) Å	θ = 3.3–25.6°
*c* = 19.7791 (10) Å	µ = 0.07 mm^−^^1^
β = 91.825 (2)°	*T* = 297 K
*V* = 1610.57 (13) Å^3^	PLATE, colourless
*Z* = 4	0.38 × 0.3 × 0.06 mm

### N-[2-(5-Methoxy-1H-indol-3-yl)ethyl]-N-propylpropan-1-amine (umd2187e_a) Data collection

**Table:**

Bruker D8 Venture CMOS diffractometer	2466 reflections with *I* > 2σ(*I*)
φ and ω scans	*R*_int_ = 0.038
Absorption correction: multi-scan (SADABS; Bruker, 2018)	θ_max_ = 25.7°, θ_min_ = 3.3°
*T*_min_ = 0.687, *T*_max_ = 0.745	*h* = −7→7
29365 measured reflections	*k* = −15→15
3035 independent reflections	*l* = −24→24

### N-[2-(5-Methoxy-1H-indol-3-yl)ethyl]-N-propylpropan-1-amine (umd2187e_a) Refinement

**Table:**

Refinement on *F*^2^	Hydrogen site location: mixed
Least-squares matrix: full	H atoms treated by a mixture of independent and constrained refinement
*R*[*F*^2^ > 2σ(*F*^2^)] = 0.042	*w* = 1/[σ^2^(*F*_o_^2^) + (0.050*P*)^2^ + 0.3694*P*] where *P* = (*F*_o_^2^ + 2*F*_c_^2^)/3
*wR*(*F*^2^) = 0.117	(Δ/σ)_max_ < 0.001
*S* = 1.06	Δρ_max_ = 0.14 e Å^−^^3^
3035 reflections	Δρ_min_ = −0.17 e Å^−^^3^
189 parameters	Extinction correction: SHELXL2018 (Sheldrick, 2015b), Fc^*^=kFc[1+0.001xFc^2^λ^3^/sin(2θ)]^-1/4^
0 restraints	Extinction coefficient: 0.046 (13)

### N-[2-(5-Methoxy-1H-indol-3-yl)ethyl]-N-propylpropan-1-amine (umd2187e_a) Special details

**Table:**

Geometry. All esds (except the esd in the dihedral angle between two l.s. planes) are estimated using the full covariance matrix. The cell esds are taken into account individually in the estimation of esds in distances, angles and torsion angles; correlations between esds in cell parameters are only used when they are defined by crystal symmetry. An approximate (isotropic) treatment of cell esds is used for estimating esds involving l.s. planes.

### N-[2-(5-Methoxy-1H-indol-3-yl)ethyl]-N-propylpropan-1-amine (umd2187e_a) Fractional atomic coordinates and isotropic or equivalent isotropic displacement parameters (Å^2^)

**Table:**

	*x*	*y*	*z*	*U*_iso_*/*U*_eq_	
O1	0.64844 (19)	0.70622 (10)	0.52927 (6)	0.0702 (4)	
N1	0.9733 (2)	0.87177 (9)	0.29883 (7)	0.0504 (3)	
N2	0.28056 (16)	0.56376 (8)	0.22986 (5)	0.0412 (3)	
C1	0.8201 (2)	0.84119 (10)	0.25168 (8)	0.0494 (4)	
H1A	0.821475	0.857658	0.205961	0.059*	
C2	0.9167 (2)	0.83449 (10)	0.36067 (7)	0.0452 (3)	
C3	1.0138 (2)	0.84559 (12)	0.42487 (8)	0.0569 (4)	
H3	1.140767	0.882380	0.430949	0.068*	
C4	0.9172 (3)	0.80093 (13)	0.47864 (8)	0.0597 (4)	
H4	0.980435	0.807252	0.521685	0.072*	
C5	0.7252 (2)	0.74592 (11)	0.47013 (8)	0.0523 (4)	
C6	0.6272 (2)	0.73433 (10)	0.40727 (7)	0.0472 (4)	
H6	0.499370	0.697976	0.401965	0.057*	
C7	0.7248 (2)	0.77873 (9)	0.35118 (7)	0.0421 (3)	
C8	0.6655 (2)	0.78344 (10)	0.28070 (7)	0.0442 (3)	
C9	0.4698 (2)	0.73615 (10)	0.24763 (8)	0.0501 (4)	
H9A	0.450938	0.763236	0.202185	0.060*	
H9B	0.344490	0.755116	0.272754	0.060*	
C10	0.4834 (2)	0.61913 (10)	0.24396 (7)	0.0429 (3)	
H10A	0.583107	0.601344	0.209131	0.051*	
H10B	0.544432	0.594539	0.286666	0.051*	
C11	0.1721 (2)	0.60049 (11)	0.16734 (7)	0.0476 (4)	
H11A	0.279545	0.613815	0.133983	0.057*	
H11B	0.100723	0.664575	0.176709	0.057*	
C12	0.0078 (2)	0.52602 (12)	0.13767 (8)	0.0530 (4)	
H12A	0.080879	0.464283	0.124003	0.064*	
H12B	−0.092372	0.507608	0.172213	0.064*	
C13	−0.1154 (4)	0.56964 (18)	0.07758 (10)	0.0941 (7)	
H13A	−0.216369	0.519855	0.060362	0.141*	
H13B	−0.017190	0.587135	0.042962	0.141*	
H13C	−0.191324	0.629728	0.091108	0.141*	
C14	0.1352 (2)	0.56727 (12)	0.28718 (7)	0.0508 (4)	
H14A	−0.007626	0.547160	0.271228	0.061*	
H14B	0.126623	0.637215	0.303036	0.061*	
C15	0.2017 (3)	0.50025 (12)	0.34569 (8)	0.0598 (4)	
H15A	0.339619	0.523201	0.364219	0.072*	
H15B	0.218942	0.430741	0.329783	0.072*	
C16	0.0390 (3)	0.50174 (17)	0.40067 (10)	0.0814 (6)	
H16A	0.084291	0.456110	0.436343	0.122*	
H16B	−0.098413	0.480319	0.382311	0.122*	
H16C	0.027543	0.569755	0.418335	0.122*	
C17	0.4531 (3)	0.65147 (16)	0.52456 (10)	0.0800 (6)	
H17A	0.414835	0.629014	0.568787	0.120*	
H17B	0.469744	0.593254	0.495692	0.120*	
H17C	0.341808	0.694924	0.505997	0.120*	
H1	1.073 (3)	0.9177 (14)	0.2916 (9)	0.066 (5)*	

### N-[2-(5-Methoxy-1H-indol-3-yl)ethyl]-N-propylpropan-1-amine (umd2187e_a) Atomic displacement parameters (Å^2^)

**Table:**

	*U*^11^	*U*^22^	*U*^33^	*U*^12^	*U*^13^	*U*^23^
O1	0.0744 (8)	0.0789 (8)	0.0573 (7)	0.0026 (6)	0.0055 (6)	0.0104 (6)
N1	0.0454 (7)	0.0430 (7)	0.0630 (8)	−0.0051 (5)	0.0051 (6)	−0.0007 (6)
N2	0.0352 (5)	0.0404 (6)	0.0482 (6)	0.0007 (4)	0.0025 (4)	0.0014 (5)
C1	0.0542 (8)	0.0387 (7)	0.0554 (8)	0.0021 (6)	0.0036 (7)	−0.0006 (6)
C2	0.0399 (7)	0.0370 (7)	0.0587 (8)	0.0038 (5)	0.0017 (6)	−0.0042 (6)
C3	0.0425 (7)	0.0584 (9)	0.0691 (10)	−0.0016 (7)	−0.0065 (7)	−0.0082 (8)
C4	0.0540 (9)	0.0680 (10)	0.0564 (9)	0.0060 (8)	−0.0074 (7)	−0.0066 (8)
C5	0.0527 (8)	0.0495 (8)	0.0550 (9)	0.0098 (7)	0.0039 (7)	0.0013 (7)
C6	0.0435 (7)	0.0394 (7)	0.0587 (9)	0.0014 (6)	0.0013 (6)	0.0002 (6)
C7	0.0387 (7)	0.0320 (6)	0.0555 (8)	0.0045 (5)	0.0010 (6)	−0.0035 (6)
C8	0.0446 (7)	0.0319 (6)	0.0559 (8)	0.0032 (5)	−0.0008 (6)	−0.0030 (6)
C9	0.0487 (8)	0.0392 (7)	0.0616 (9)	0.0025 (6)	−0.0092 (6)	−0.0008 (6)
C10	0.0363 (7)	0.0395 (7)	0.0528 (8)	0.0019 (5)	0.0013 (5)	−0.0017 (6)
C11	0.0469 (7)	0.0464 (8)	0.0493 (8)	−0.0055 (6)	−0.0007 (6)	0.0040 (6)
C12	0.0456 (8)	0.0514 (8)	0.0617 (9)	−0.0052 (6)	−0.0030 (6)	0.0013 (7)
C13	0.0982 (15)	0.1021 (16)	0.0794 (13)	−0.0435 (13)	−0.0369 (11)	0.0253 (12)
C14	0.0422 (7)	0.0563 (9)	0.0543 (8)	0.0048 (6)	0.0060 (6)	0.0050 (7)
C15	0.0695 (10)	0.0524 (9)	0.0579 (9)	0.0011 (8)	0.0090 (7)	0.0071 (7)
C16	0.0866 (13)	0.0960 (15)	0.0627 (11)	−0.0025 (11)	0.0185 (9)	0.0148 (10)
C17	0.0822 (12)	0.0812 (13)	0.0773 (12)	−0.0058 (10)	0.0137 (10)	0.0226 (10)

### N-[2-(5-Methoxy-1H-indol-3-yl)ethyl]-N-propylpropan-1-amine (umd2187e_a) Geometric parameters (Å, º)

**Table:**

O1—C5	1.3794 (18)	C10—H10A	0.9700
O1—C17	1.412 (2)	C10—H10B	0.9700
N1—C1	1.3716 (19)	C11—H11A	0.9700
N1—C2	1.3734 (19)	C11—H11B	0.9700
N1—H1	0.878 (18)	C11—C12	1.5171 (19)
N2—C10	1.4745 (16)	C12—H12A	0.9700
N2—C11	1.4705 (17)	C12—H12B	0.9700
N2—C14	1.4735 (17)	C12—C13	1.506 (2)
C1—H1A	0.9300	C13—H13A	0.9600
C1—C8	1.364 (2)	C13—H13B	0.9600
C2—C3	1.396 (2)	C13—H13C	0.9600
C2—C7	1.4070 (18)	C14—H14A	0.9700
C3—H3	0.9300	C14—H14B	0.9700
C3—C4	1.369 (2)	C14—C15	1.500 (2)
C4—H4	0.9300	C15—H15A	0.9700
C4—C5	1.401 (2)	C15—H15B	0.9700
C5—C6	1.376 (2)	C15—C16	1.510 (2)
C6—H6	0.9300	C16—H16A	0.9600
C6—C7	1.4071 (19)	C16—H16B	0.9600
C7—C8	1.4318 (19)	C16—H16C	0.9600
C8—C9	1.4980 (19)	C17—H17A	0.9600
C9—H9A	0.9700	C17—H17B	0.9600
C9—H9B	0.9700	C17—H17C	0.9600
C9—C10	1.5363 (18)		
			
C5—O1—C17	117.15 (13)	N2—C11—H11A	108.8
C1—N1—C2	108.02 (12)	N2—C11—H11B	108.8
C1—N1—H1	124.6 (11)	N2—C11—C12	113.62 (11)
C2—N1—H1	126.2 (11)	H11A—C11—H11B	107.7
C11—N2—C10	111.44 (10)	C12—C11—H11A	108.8
C11—N2—C14	111.12 (10)	C12—C11—H11B	108.8
C14—N2—C10	112.44 (11)	C11—C12—H12A	109.1
N1—C1—H1A	124.5	C11—C12—H12B	109.1
C8—C1—N1	111.08 (13)	H12A—C12—H12B	107.9
C8—C1—H1A	124.5	C13—C12—C11	112.43 (13)
N1—C2—C3	131.08 (13)	C13—C12—H12A	109.1
N1—C2—C7	107.83 (12)	C13—C12—H12B	109.1
C3—C2—C7	121.08 (14)	C12—C13—H13A	109.5
C2—C3—H3	120.8	C12—C13—H13B	109.5
C4—C3—C2	118.30 (14)	C12—C13—H13C	109.5
C4—C3—H3	120.8	H13A—C13—H13B	109.5
C3—C4—H4	119.3	H13A—C13—H13C	109.5
C3—C4—C5	121.32 (15)	H13B—C13—H13C	109.5
C5—C4—H4	119.3	N2—C14—H14A	108.6
O1—C5—C4	114.14 (14)	N2—C14—H14B	108.6
C6—C5—O1	124.69 (14)	N2—C14—C15	114.48 (12)
C6—C5—C4	121.16 (14)	H14A—C14—H14B	107.6
C5—C6—H6	120.8	C15—C14—H14A	108.6
C5—C6—C7	118.48 (13)	C15—C14—H14B	108.6
C7—C6—H6	120.8	C14—C15—H15A	109.2
C2—C7—C6	119.65 (13)	C14—C15—H15B	109.2
C2—C7—C8	107.37 (12)	C14—C15—C16	111.92 (14)
C6—C7—C8	132.94 (12)	H15A—C15—H15B	107.9
C1—C8—C7	105.68 (12)	C16—C15—H15A	109.2
C1—C8—C9	128.30 (14)	C16—C15—H15B	109.2
C7—C8—C9	126.00 (13)	C15—C16—H16A	109.5
C8—C9—H9A	109.0	C15—C16—H16B	109.5
C8—C9—H9B	109.0	C15—C16—H16C	109.5
C8—C9—C10	112.80 (11)	H16A—C16—H16B	109.5
H9A—C9—H9B	107.8	H16A—C16—H16C	109.5
C10—C9—H9A	109.0	H16B—C16—H16C	109.5
C10—C9—H9B	109.0	O1—C17—H17A	109.5
N2—C10—C9	116.81 (11)	O1—C17—H17B	109.5
N2—C10—H10A	108.1	O1—C17—H17C	109.5
N2—C10—H10B	108.1	H17A—C17—H17B	109.5
C9—C10—H10A	108.1	H17A—C17—H17C	109.5
C9—C10—H10B	108.1	H17B—C17—H17C	109.5
H10A—C10—H10B	107.3		
			
O1—C5—C6—C7	−179.67 (13)	C3—C4—C5—C6	0.3 (2)
N1—C1—C8—C7	0.81 (15)	C4—C5—C6—C7	0.3 (2)
N1—C1—C8—C9	179.30 (13)	C5—C6—C7—C2	−0.84 (19)
N1—C2—C3—C4	178.54 (14)	C5—C6—C7—C8	−178.36 (13)
N1—C2—C7—C6	−178.18 (11)	C6—C7—C8—C1	177.31 (14)
N1—C2—C7—C8	−0.08 (14)	C6—C7—C8—C9	−1.2 (2)
N2—C11—C12—C13	174.55 (15)	C7—C2—C3—C4	−0.1 (2)
N2—C14—C15—C16	−176.15 (14)	C7—C8—C9—C10	−71.42 (18)
C1—N1—C2—C3	−178.24 (14)	C8—C9—C10—N2	163.85 (12)
C1—N1—C2—C7	0.57 (15)	C10—N2—C11—C12	160.69 (11)
C1—C8—C9—C10	110.38 (16)	C10—N2—C14—C15	−74.13 (16)
C2—N1—C1—C8	−0.88 (16)	C11—N2—C10—C9	55.06 (16)
C2—C3—C4—C5	−0.4 (2)	C11—N2—C14—C15	160.17 (13)
C2—C7—C8—C1	−0.44 (14)	C14—N2—C10—C9	−70.46 (15)
C2—C7—C8—C9	−178.97 (12)	C14—N2—C11—C12	−73.06 (15)
C3—C2—C7—C6	0.77 (19)	C17—O1—C5—C4	178.83 (15)
C3—C2—C7—C8	178.88 (12)	C17—O1—C5—C6	−1.2 (2)
C3—C4—C5—O1	−179.69 (14)		

### N-[2-(5-Methoxy-1H-indol-3-yl)ethyl]-N-propylpropan-1-amine (umd2187e_a) Hydrogen-bond geometry (Å, º)

**Table:**

*D*—H···*A*	*D*—H	H···*A*	*D*···*A*	*D*—H···*A*
N1—H1···N2^i^	0.878 (18)	2.167 (19)	3.0070 (17)	160.0 (16)

Symmetry code: (i) −*x*+3/2, *y*+1/2, −*z*+1/2.

### Bis{N-[2-(5-methoxy-1H-indol-3-yl)ethyl]-N-propylpropan-1-aminium}; but-2-enedioate (umd2188f_a) Crystal data

**Table:**

C_17_H_27_N_2_O^+^·0.5C_4_H_2_O_4_^2^^−^	*Z* = 2
*M**_r_* = 332.43	*F*(000) = 360
Triclinic, *P*1	*D*_x_ = 1.171 Mg m^−^^3^
*a* = 9.2956 (6) Å	Mo *K*α radiation, λ = 0.71073 Å
*b* = 9.4443 (6) Å	Cell parameters from 9966 reflections
*c* = 12.7427 (8) Å	θ = 2.6–25.6°
α = 78.552 (2)°	µ = 0.08 mm^−^^1^
β = 75.929 (2)°	*T* = 297 K
γ = 60.806 (2)°	BLOCK, colourless
*V* = 943.06 (11) Å^3^	0.3 × 0.22 × 0.2 mm

### Bis{N-[2-(5-methoxy-1H-indol-3-yl)ethyl]-N-propylpropan-1-aminium}; but-2-enedioate (umd2188f_a) Data collection

**Table:**

Bruker D8 Venture CMOS diffractometer	3006 reflections with *I* > 2σ(*I*)
φ and ω scans	*R*_int_ = 0.032
Absorption correction: multi-scan (SADABS; Bruker, 2018)	θ_max_ = 25.8°, θ_min_ = 2.6°
*T*_min_ = 0.722, *T*_max_ = 0.745	*h* = −11→11
37231 measured reflections	*k* = −11→11
3565 independent reflections	*l* = −15→15

### Bis{N-[2-(5-methoxy-1H-indol-3-yl)ethyl]-N-propylpropan-1-aminium}; but-2-enedioate (umd2188f_a) Refinement

**Table:**

Refinement on *F*^2^	0 restraints
Least-squares matrix: full	Hydrogen site location: mixed
*R*[*F*^2^ > 2σ(*F*^2^)] = 0.052	H atoms treated by a mixture of independent and constrained refinement
*wR*(*F*^2^) = 0.142	*w* = 1/[σ^2^(*F*_o_^2^) + (0.0652*P*)^2^ + 0.3068*P*] where *P* = (*F*_o_^2^ + 2*F*_c_^2^)/3
*S* = 1.05	(Δ/σ)_max_ < 0.001
3565 reflections	Δρ_max_ = 0.29 e Å^−^^3^
228 parameters	Δρ_min_ = −0.15 e Å^−^^3^

### Bis{N-[2-(5-methoxy-1H-indol-3-yl)ethyl]-N-propylpropan-1-aminium}; but-2-enedioate (umd2188f_a) Special details

**Table:**

Geometry. All esds (except the esd in the dihedral angle between two l.s. planes) are estimated using the full covariance matrix. The cell esds are taken into account individually in the estimation of esds in distances, angles and torsion angles; correlations between esds in cell parameters are only used when they are defined by crystal symmetry. An approximate (isotropic) treatment of cell esds is used for estimating esds involving l.s. planes.

### Bis{N-[2-(5-methoxy-1H-indol-3-yl)ethyl]-N-propylpropan-1-aminium}; but-2-enedioate (umd2188f_a) Fractional atomic coordinates and isotropic or equivalent isotropic displacement parameters (Å^2^)

**Table:**

	*x*	*y*	*z*	*U*_iso_*/*U*_eq_	
O1	1.12338 (18)	0.1921 (2)	0.42565 (12)	0.0820 (5)	
O3	0.56136 (18)	0.70109 (14)	0.91994 (11)	0.0619 (4)	
O4	0.38521 (19)	0.94777 (15)	0.85790 (10)	0.0619 (4)	
N1	0.6457 (2)	0.7968 (2)	0.29933 (12)	0.0592 (4)	
N2	0.45383 (17)	0.62296 (15)	0.77576 (10)	0.0428 (3)	
C1	0.5216 (3)	0.8238 (2)	0.38795 (14)	0.0569 (5)	
H1A	0.419516	0.917793	0.395103	0.068*	
C2	0.7767 (2)	0.6468 (2)	0.31704 (13)	0.0494 (4)	
C3	0.9290 (3)	0.5609 (3)	0.25118 (14)	0.0611 (5)	
H3	0.955315	0.605071	0.181743	0.073*	
C4	1.0389 (2)	0.4104 (3)	0.29047 (15)	0.0635 (5)	
H4	1.140948	0.351877	0.247161	0.076*	
C5	1.0004 (2)	0.3427 (2)	0.39518 (15)	0.0564 (4)	
C6	0.8487 (2)	0.4243 (2)	0.45998 (13)	0.0497 (4)	
H6	0.822409	0.377642	0.528497	0.060*	
C7	0.7346 (2)	0.5788 (2)	0.42102 (12)	0.0450 (4)	
C8	0.5686 (2)	0.6934 (2)	0.46471 (13)	0.0474 (4)	
C9	0.4679 (2)	0.6678 (2)	0.57159 (13)	0.0477 (4)	
H9A	0.480231	0.558342	0.579746	0.057*	
H9B	0.350672	0.743536	0.570911	0.057*	
C10	0.5191 (2)	0.6913 (2)	0.66834 (12)	0.0470 (4)	
H10A	0.640253	0.639174	0.658321	0.056*	
H10B	0.478562	0.806815	0.670881	0.056*	
C11	0.2669 (2)	0.7020 (2)	0.79912 (13)	0.0506 (4)	
H11A	0.228284	0.658211	0.755537	0.061*	
H11B	0.223555	0.817953	0.777702	0.061*	
C12	0.1977 (3)	0.6766 (3)	0.91717 (16)	0.0767 (6)	
H12A	0.232415	0.561287	0.936852	0.092*	
H12B	0.244506	0.711049	0.961289	0.092*	
C13	0.0122 (3)	0.7678 (4)	0.9418 (2)	0.1035 (10)	
H13A	−0.026624	0.732314	1.013936	0.155*	
H13B	−0.034648	0.747832	0.890459	0.155*	
H13C	−0.021980	0.882313	0.937002	0.155*	
C14	0.5291 (2)	0.44102 (19)	0.78992 (14)	0.0502 (4)	
H14A	0.504193	0.405008	0.733484	0.060*	
H14B	0.477479	0.408059	0.859428	0.060*	
C15	0.7152 (2)	0.3582 (2)	0.78522 (16)	0.0616 (5)	
H15A	0.740808	0.395586	0.840557	0.074*	
H15B	0.767606	0.388253	0.714986	0.074*	
C16	0.7866 (3)	0.1758 (3)	0.8025 (2)	0.0940 (8)	
H16A	0.906071	0.127238	0.795009	0.141*	
H16B	0.758181	0.138666	0.749425	0.141*	
H16C	0.741153	0.145089	0.874057	0.141*	
C17	1.1057 (3)	0.1353 (3)	0.5365 (2)	0.0943 (8)	
H17A	1.209504	0.044150	0.551443	0.141*	
H17B	1.019527	0.101981	0.553796	0.141*	
H17C	1.076098	0.220867	0.579927	0.141*	
C18	0.4904 (2)	0.85411 (19)	0.91702 (12)	0.0450 (4)	
C19	0.5356 (2)	0.92260 (19)	0.99289 (13)	0.0459 (4)	
H19	0.619664	0.850477	1.032462	0.055*	
H2	0.488 (3)	0.653 (3)	0.8317 (18)	0.076 (6)*	
H1	0.632 (3)	0.872 (3)	0.247 (2)	0.082 (7)*	

### Bis{N-[2-(5-methoxy-1H-indol-3-yl)ethyl]-N-propylpropan-1-aminium}; but-2-enedioate (umd2188f_a) Atomic displacement parameters (Å^2^)

**Table:**

	*U*^11^	*U*^22^	*U*^33^	*U*^12^	*U*^13^	*U*^23^
O1	0.0612 (9)	0.0809 (10)	0.0683 (9)	−0.0035 (7)	−0.0085 (7)	−0.0156 (8)
O3	0.0872 (9)	0.0419 (7)	0.0638 (8)	−0.0273 (6)	−0.0301 (7)	−0.0058 (5)
O4	0.0964 (10)	0.0497 (7)	0.0545 (7)	−0.0402 (7)	−0.0335 (7)	0.0072 (5)
N1	0.0791 (11)	0.0579 (9)	0.0412 (8)	−0.0367 (9)	−0.0112 (7)	0.0089 (7)
N2	0.0549 (8)	0.0428 (7)	0.0346 (6)	−0.0256 (6)	−0.0093 (6)	−0.0018 (5)
C1	0.0699 (12)	0.0494 (10)	0.0460 (9)	−0.0234 (9)	−0.0121 (8)	−0.0018 (7)
C2	0.0618 (10)	0.0595 (10)	0.0377 (8)	−0.0384 (9)	−0.0076 (7)	0.0000 (7)
C3	0.0678 (12)	0.0912 (15)	0.0387 (9)	−0.0523 (11)	0.0002 (8)	−0.0052 (9)
C4	0.0516 (10)	0.0923 (15)	0.0507 (10)	−0.0368 (11)	0.0031 (8)	−0.0204 (10)
C5	0.0494 (10)	0.0653 (11)	0.0528 (10)	−0.0225 (9)	−0.0094 (8)	−0.0125 (8)
C6	0.0530 (9)	0.0553 (10)	0.0389 (8)	−0.0253 (8)	−0.0057 (7)	−0.0027 (7)
C7	0.0528 (9)	0.0508 (9)	0.0366 (8)	−0.0290 (8)	−0.0062 (7)	−0.0034 (6)
C8	0.0589 (10)	0.0483 (9)	0.0382 (8)	−0.0269 (8)	−0.0091 (7)	−0.0039 (6)
C9	0.0522 (9)	0.0518 (9)	0.0401 (8)	−0.0246 (8)	−0.0066 (7)	−0.0069 (7)
C10	0.0602 (10)	0.0447 (8)	0.0402 (8)	−0.0278 (8)	−0.0094 (7)	−0.0021 (6)
C11	0.0560 (10)	0.0518 (9)	0.0417 (8)	−0.0234 (8)	−0.0094 (7)	−0.0020 (7)
C12	0.0666 (13)	0.0915 (16)	0.0490 (11)	−0.0273 (12)	−0.0023 (9)	0.0073 (10)
C13	0.0757 (16)	0.114 (2)	0.0622 (14)	−0.0133 (15)	0.0082 (12)	0.0053 (13)
C14	0.0616 (10)	0.0422 (9)	0.0496 (9)	−0.0266 (8)	−0.0120 (8)	0.0001 (7)
C15	0.0619 (11)	0.0588 (11)	0.0547 (10)	−0.0233 (9)	−0.0086 (8)	0.0003 (8)
C16	0.0797 (16)	0.0574 (13)	0.1040 (19)	−0.0150 (12)	0.0017 (14)	0.0112 (12)
C17	0.0861 (17)	0.0747 (15)	0.0759 (16)	0.0002 (13)	−0.0186 (13)	−0.0065 (12)
C18	0.0623 (10)	0.0445 (9)	0.0353 (7)	−0.0316 (8)	−0.0082 (7)	−0.0003 (6)
C19	0.0571 (10)	0.0450 (8)	0.0415 (8)	−0.0280 (7)	−0.0102 (7)	−0.0024 (6)

### Bis{N-[2-(5-methoxy-1H-indol-3-yl)ethyl]-N-propylpropan-1-aminium}; but-2-enedioate (umd2188f_a) Geometric parameters (Å, º)

**Table:**

O1—C5	1.375 (2)	C10—H10A	0.9700
O1—C17	1.408 (3)	C10—H10B	0.9700
O3—C18	1.2592 (19)	C11—H11A	0.9700
O4—C18	1.247 (2)	C11—H11B	0.9700
N1—C1	1.365 (2)	C11—C12	1.507 (2)
N1—C2	1.365 (2)	C12—H12A	0.9700
N1—H1	0.86 (2)	C12—H12B	0.9700
N2—C10	1.512 (2)	C12—C13	1.486 (3)
N2—C11	1.496 (2)	C13—H13A	0.9600
N2—C14	1.497 (2)	C13—H13B	0.9600
N2—H2	0.98 (2)	C13—H13C	0.9600
C1—H1A	0.9300	C14—H14A	0.9700
C1—C8	1.367 (2)	C14—H14B	0.9700
C2—C3	1.392 (3)	C14—C15	1.503 (3)
C2—C7	1.406 (2)	C15—H15A	0.9700
C3—H3	0.9300	C15—H15B	0.9700
C3—C4	1.365 (3)	C15—C16	1.504 (3)
C4—H4	0.9300	C16—H16A	0.9600
C4—C5	1.404 (3)	C16—H16B	0.9600
C5—C6	1.375 (2)	C16—H16C	0.9600
C6—H6	0.9300	C17—H17A	0.9600
C6—C7	1.400 (2)	C17—H17B	0.9600
C7—C8	1.433 (2)	C17—H17C	0.9600
C8—C9	1.503 (2)	C18—C19	1.497 (2)
C9—H9A	0.9700	C19—C19^i^	1.308 (3)
C9—H9B	0.9700	C19—H19	0.9300
C9—C10	1.514 (2)		
			
C5—O1—C17	116.90 (16)	N2—C11—H11B	109.0
C1—N1—H1	119.0 (17)	N2—C11—C12	113.04 (14)
C2—N1—C1	108.73 (15)	H11A—C11—H11B	107.8
C2—N1—H1	132.3 (17)	C12—C11—H11A	109.0
C10—N2—H2	105.8 (13)	C12—C11—H11B	109.0
C11—N2—C10	112.05 (12)	C11—C12—H12A	109.0
C11—N2—C14	111.85 (13)	C11—C12—H12B	109.0
C11—N2—H2	106.2 (13)	H12A—C12—H12B	107.8
C14—N2—C10	114.43 (12)	C13—C12—C11	112.83 (18)
C14—N2—H2	105.8 (13)	C13—C12—H12A	109.0
N1—C1—H1A	124.8	C13—C12—H12B	109.0
N1—C1—C8	110.45 (17)	C12—C13—H13A	109.5
C8—C1—H1A	124.8	C12—C13—H13B	109.5
N1—C2—C3	131.24 (16)	C12—C13—H13C	109.5
N1—C2—C7	107.90 (15)	H13A—C13—H13B	109.5
C3—C2—C7	120.85 (17)	H13A—C13—H13C	109.5
C2—C3—H3	120.6	H13B—C13—H13C	109.5
C4—C3—C2	118.74 (16)	N2—C14—H14A	108.9
C4—C3—H3	120.6	N2—C14—H14B	108.9
C3—C4—H4	119.5	N2—C14—C15	113.16 (14)
C3—C4—C5	121.06 (17)	H14A—C14—H14B	107.8
C5—C4—H4	119.5	C15—C14—H14A	108.9
O1—C5—C4	115.19 (17)	C15—C14—H14B	108.9
O1—C5—C6	123.99 (17)	C14—C15—H15A	109.2
C6—C5—C4	120.82 (18)	C14—C15—H15B	109.2
C5—C6—H6	120.6	C14—C15—C16	111.96 (18)
C5—C6—C7	118.85 (16)	H15A—C15—H15B	107.9
C7—C6—H6	120.6	C16—C15—H15A	109.2
C2—C7—C8	106.94 (15)	C16—C15—H15B	109.2
C6—C7—C2	119.63 (15)	C15—C16—H16A	109.5
C6—C7—C8	133.41 (15)	C15—C16—H16B	109.5
C1—C8—C7	105.96 (15)	C15—C16—H16C	109.5
C1—C8—C9	128.30 (17)	H16A—C16—H16B	109.5
C7—C8—C9	125.70 (15)	H16A—C16—H16C	109.5
C8—C9—H9A	108.9	H16B—C16—H16C	109.5
C8—C9—H9B	108.9	O1—C17—H17A	109.5
C8—C9—C10	113.31 (14)	O1—C17—H17B	109.5
H9A—C9—H9B	107.7	O1—C17—H17C	109.5
C10—C9—H9A	108.9	H17A—C17—H17B	109.5
C10—C9—H9B	108.9	H17A—C17—H17C	109.5
N2—C10—C9	113.69 (13)	H17B—C17—H17C	109.5
N2—C10—H10A	108.8	O3—C18—C19	115.90 (14)
N2—C10—H10B	108.8	O4—C18—O3	124.28 (15)
C9—C10—H10A	108.8	O4—C18—C19	119.80 (14)
C9—C10—H10B	108.8	C18—C19—H19	117.9
H10A—C10—H10B	107.7	C19^i^—C19—C18	124.2 (2)
N2—C11—H11A	109.0	C19^i^—C19—H19	117.9
			
O1—C5—C6—C7	178.60 (17)	C3—C4—C5—O1	−178.61 (17)
O3—C18—C19—C19^i^	−174.3 (2)	C3—C4—C5—C6	1.8 (3)
O4—C18—C19—C19^i^	4.2 (3)	C4—C5—C6—C7	−1.9 (3)
N1—C1—C8—C7	1.0 (2)	C5—C6—C7—C2	0.2 (2)
N1—C1—C8—C9	−176.59 (16)	C5—C6—C7—C8	178.52 (17)
N1—C2—C3—C4	179.92 (18)	C6—C7—C8—C1	179.95 (18)
N1—C2—C7—C6	−179.66 (15)	C6—C7—C8—C9	−2.4 (3)
N1—C2—C7—C8	1.61 (18)	C7—C2—C3—C4	−1.6 (3)
N2—C11—C12—C13	174.7 (2)	C7—C8—C9—C10	78.1 (2)
N2—C14—C15—C16	178.56 (17)	C8—C9—C10—N2	−164.36 (13)
C1—N1—C2—C3	177.59 (18)	C10—N2—C11—C12	−162.09 (16)
C1—N1—C2—C7	−1.0 (2)	C10—N2—C14—C15	60.72 (19)
C1—C8—C9—C10	−104.8 (2)	C11—N2—C10—C9	−60.66 (18)
C2—N1—C1—C8	0.0 (2)	C11—N2—C14—C15	−170.49 (14)
C2—C3—C4—C5	0.0 (3)	C14—N2—C10—C9	68.03 (18)
C2—C7—C8—C1	−1.58 (19)	C14—N2—C11—C12	67.9 (2)
C2—C7—C8—C9	176.07 (15)	C17—O1—C5—C4	166.9 (2)
C3—C2—C7—C6	1.5 (2)	C17—O1—C5—C6	−13.5 (3)
C3—C2—C7—C8	−177.18 (15)		

Symmetry code: (i) −*x*+1, −*y*+2, −*z*+2.

### Bis{N-[2-(5-methoxy-1H-indol-3-yl)ethyl]-N-propylpropan-1-aminium}; but-2-enedioate (umd2188f_a) Hydrogen-bond geometry (Å, º)

**Table:**

*D*—H···*A*	*D*—H	H···*A*	*D*···*A*	*D*—H···*A*
N2—H2···O3	0.98 (2)	1.68 (2)	2.6588 (17)	175 (2)
N1—H1···O4^ii^	0.86 (2)	1.91 (3)	2.757 (2)	171 (2)

Symmetry code: (ii) −*x*+1, −*y*+2, −*z*+1.
